# American Medical Authors and Library of the Royal College of Surgeons, England

**Published:** 1856-09

**Authors:** Austin Flint


### *American Medical Authors and Library of the Royal College of Surgeons, England.*

Dear Doctor: I beg leave, through the medium of your columns, to fulfill a promise too long neglected.

During my brief stay in London, in August, 1854, it was my good fortune to become acquainted with Mr. John Chatto, a medical writer well known by his able contributions to the British and Foreign Medico-Chirurgical Review. Mr. C. was at that time, and I presume is still, librarian to the Royal College of Surgeons. This medical library, contained in the college edifice which also contains the Hunterian Museum, is, I believe, the largest in London, embracing works on all the departments of medical science, being, as I was told, much more extensive than the library of the Royal College of Physicians.

In my conversations with Mr. C. I was not less gratified than surprised to find that he was minutely and accurately informed concerning American medical works, and he expressed to me a great desire that copies of new publications should be sent to the library over which he has charge. He wished it to be known to authors on this side of the Atlantic, that their works would be received with great pleasure, handsomely bound, placed conspicuously in the library and catalogued. I promised on my return to take some pains to circulate this information. I postponed the fulfillment of this promise for some time, intending to add to the series of letters which I wrote from Paris, one or more, giving some account of medical matters coming under my observation in Great Britain. So long a period has now elapsed, that I have no expectation of carrying out this intention, and I have therefore, thus tardily, embraced the present method of stating to your readers what I should have introduced into a letter from London had the latter been written.

Truly yours,

Austin Flint.

